# Supplementary material for: Reprograming of Gene Expression of Key Inflammatory Signaling Pathways in Human Peripheral Blood Mononuclear Cells by Soybean Lectin and Resveratrol
Source: Int J Mol Sci. 2022 Oct 26;23(21):12946. doi: 10.3390/ijms232112946 (PMC9659230; doi:10.3390/ijms232112946)
Supplement: Supplementary file 1 [file ijms-23-12946-s001.zip › ijms-1917909-supplementary.pdf]

SUPPLEMENTARY MATERIALS

TABLE S1. GENES UPREGULATED BY RESVERATROL ALONE*.

RES	Control	log2 Fold	*P* value	Gene Name	Gene Description
6.825	0.265	4.648	$1.45x10^{-12}$	ITGAD	Integrin-alpha D
46.964	9.570	2.294	$2.33\ x10^{-07}$	CTC-205M6.5	-
1687.47	414.500	2.025	$2.00\ x10^{-06}$	NEAT1	nuclear_paraspeckle_assembly_transcript_1_(non-protein coding)
33.109	7.7625	2.091	$2.54\ x10^{-06}$	TMEM119	transmembrane_protein_119

*These data represent the genes that are upregulated by resveratrol alone as compared to a vehicle control in PBMCs (incubated for 3h). RNAs were extracted and subjected to RNAseq analysis. This data has been obtained by using the DEG program.

TABLE S2. (A) LPS-INDUCED GENES MODULATED BY RESVERATROL*. (B) LPS-INDUCED GENES NOT MODULATED BY RESVERATROL**. (C) DOWN-REGULATED LPS MODULATED GENES NOT AFFECTED BY RESVERATROL**.

A.

LPS + RES	LPS	Log2 Fold	*P* value	Gene Name	Gene Description
0.0529	9.74207	-7.3514	$1.41\ x10^{-18}$	CCL8	chemokine_(C-C_motif)_ligand_8
27.447	476.382	-4.1171	$1.49\ x10^{-18}$	CYP1B1	cytochrome_P450_family_1_subfamily_B_polypeptide_1
7.7361	135.790	-4.1325	$4.13\ x10^{-18}$	CCL2	chemokine_(C-C_motif)_ligand_2
125.420	1442.97	-3.5241	$7.36\ x10^{-15}$	RSAD2	radical_S-adenosyl_methionine_domain_containing_2
4.715	61.064	-3.6929	$8.29\ x10^{-15}$	RGL1	ral_guanine_nucleotide_dissociation_stimulator-like_1
8.954	108.360	-3.596	$1.04\ x10^{-14}$	CCL7	chemokine_(C-C_motif)_ligand_7
62.895	689.129	-3.453	$2.18\ x10^{-14}$	OAS3	2'-5'-oligoadenylate_synthetase_3_100kDa
0.105	6.422	-5.834	$5.39\ x10^{-14}$	IL19	interleukin_19
0.264	8.381	-4.948	$5.55\ x10^{-14}$	CYP1B1-AS1	CYP1B1_antisense_RNA_1
57.067	593.613	-3.378	$6.55\ x10^{-14}$	CMPK2	cytidine_monophosphate_(UMP-CMP) kinase_2_mitochondrial
5.0337	54.642	-3.438	$2.76\ x10^{-13}$	CXCL11	chemokine_(C-X-C_motif)_ligand_11
6.358	63.133	-3.310	$9.45\ x10^{-13}$	IFNG	interferon_gamma
2.331	25.960	-3.473	$1.33\ x10^{-12}$	SIGLEC1	sialic_acid_binding_Ig-like_lectin_1_sialoadhesin
0.688	11.102	-3.997	$1.61\ x10^{-12}$	GGT5	gamma-glutamyltransferase_5
13.988	118.319	-3.079	$8.84\ x10^{-12}$	ARHGEF 11	Rho_guanine_nucleotide_exchange_factor_(GEF)_11
23.526	194.569	-3.047	$1.01\ x10^{-11}$	NEU4	sialidase_4
0.635	9.252	-3.849	$2.12\ x10^{-11}$	IL10	interleukin_10
1.006	12.136	-3.582	$2.65\ x10^{-11}$	EDN1	endothelin_1
6.093	50.397	-3.046	$3.61\ x10^{-11}$	CSF2	colony_stimulating_factor_2_(granulocyte-macrophage)
17.538	134.484	-2.938	$5.35\ x10^{-11}$	USP18	ubiquitin_specific_peptidase_18
5.351	43.920	-3.035	$5.93\ x10^{-11}$	EGR2	early_growth_response_2
61.358	438.99	-2.838	$1.16\ x10^{-10}$	SAMD9L	sterile_alpha_motif_domain_containing_9-like
0.105	3.973	-5.142	$2.01\ x10^{-10}$	LAMA2	laminin_alpha_2
1023.76	6937.936	-2.760	$2.90\ x10\text{-}^{10}$	THBS1	thrombospondin_1
171.677	1129.917	-2.718	$5.22\ x10^{-10}$	MX1	myxovirus_(influenza_virus)_resistance_1_interferon-inducible_protein_p78_(mouse)
237.487	1548.772	-2.705	$6.12\ x10^{-10}$	HELZ2	helicase_with_zinc_finger_2_transcriptional_coactivator
0.635	7.510	-3.548	$7.95\ x10^{-10}$	MACC1	metastasis_associated_in_colon_cancer_1

3.020	22.804	-2.913	9.03 x10^{-10}	SPINK1	serine_peptidase_inhibitor_Kazal_type_1
28.560	181.235	-2.665	1.44 x10^{-09}	PNPT1	polyribonucleotide_nucleotidyltransferase_1
0.688	7.565	-3.444	1.57 x10^{-09}	PNPLA1	patatin-like_phospholipase_domain_containing_1
30.255	4.571	2.724	3.76 x10^{-09}	TMEM119	transmembrane_protein_119
51.026	302.657	-2.568	3.92 x10^{-09}	CXCL10	chemokine_(C-X-C_motif)_ligand_10
104.331	610.920	-2.549	4.59 x10^{-09}	SLC39A8	solute_carrier_family_39_(zinc_transporter)_member_8
68.035	395.343	-2.538	5.47 x10^{-09}	EIF2AK2	eukaryotic_translation_initiation_factor_2-alpha_kinase_2
4.450	28.301	-2.666	8.53 x10^{-09}	ETV7	ets_variant_7
3.073	20.191	-2.713	1.02 x10^{-08}	MGAM	maltase-glucoamylase_(alpha-glucosidase)
2.1724	0	8.342	1.09 x10^{-08}	DKK2	dickkopf_2_homolog_(Xenopus_laevis)
14.7833	84.1954	-2.509	1.36 x10^{-08}	IFI44L	interferon-induced_protein_44-like
1.69558	11.9190	-2.808	1.76 x10^{-08}	NEXN	nexilin_(F_actin_binding_protein)
1.43064	10.6128	-2.885	2.07 x10^{-08}	INHBA-AS1	INHBA_antisense_RNA_1
106.503	560.359	-2.395	3.04 x10^{-08}	INHBA	inhibin_beta_A
37.832	196.474	-2.376	4.55 x10^{-08}	OAS1	2'-5'-oligoadenylate_synthetase_1_40/46kDa
6.305	34.940	-2.469	4.72 x10^{-08}	PDGFB	platelet-derived_growth_factor_beta_polypeptide
0.847	6.966	-3.028	4.92 x10^{-08}	ABCA6	ATP-binding_cassette_sub-family_A_(ABC1)_member_6
8.371	1.142	2.865	5.13 x10^{-08}	C2CD4B	C2_calcium-dependent_domain_containing_4B
2.914	17.361	-2.571	6.05 x10^{-08}	IL36G	interleukin_36_gamma
26.705	136.552	-2.354	6.60 x10^{-08}	NCOA7	nuclear_receptor_coactivator_7
0.847	6.803	-2.994	7.09 x10^{-08}	BATF2	basic_leucine_zipper_transcription_factor_ATF-like_2
3.921	22.314	-2.506	7.16 x10^{-08}	MLKL	mixed_lineage_kinase_domain-like
4.185	0.380	3.434	8.61 x10^{-08}	WDR78	WD_repeat_domain_78
74.128	364.212	-2.296	1.01 x10^{-07}	IFI6	interferon_alpha-inducible_protein_6
0.211	3.211	-3.879	1.12 x10^{-07}	GCNT2	glucosaminyl_(N-acetyl)_transferase_2_I-branching_enzyme_(I_blood_group)
17.379	3.047	2.509	1.22 x10^{-07}	SLC9A7P1	solute_carrier_family_9_subfamily_A_(NHE7_cation_proton_antiporter_7)_member_7_pseudogene_1
0.317	3.755	-3.534	1.42 x10^{-07}	LEKR1	leucine_glutamate_and_lysine_rich_1
148.575	709.865	-2.256	1.55 x10^{-07}	SLC7A11	solute_carrier_family_7_(anionic_amino_acid_transporter_light_chain_xc-system)_member_11
52.404	251.11	-2.260	1.61 x10^{-07}	HERC6	HECT_and_RLD_domain_containing_E3_ubiquitin_protein_ligase_family_member_6
2.331	13.442	-2.524	1.76 x10^{-07}	CACNA1A	calcium_channel_voltage-dependent_P/Q_type_alpha_1A_subunit
2.702	14.912	-2.461	2.39 x10^{-07}	CXCL9	chemokine_(C-X-C_motif)_ligand_9
0	1.741	-8.025	2.40 x10^{-07}	KRT8P42	keratin_8_pseudogene_42
59.080	275.444	-2.220	2.50 x10^{-07}	DHX58	DEXH_(Asp-Glu-X-His)_box_polypeptide_58
0.794	6.041	-2.915	2.53 x10^{-07}	IL27	interleukin_27
18.810	89.420	-2.248	2.57 x10^{-07}	CD93	CD93_molecule
2.755	15.075	-2.449	2.63 x10^{-07}	SYNPO2	synaptopodin_2
0.105	2.394	-4.413	2.67 x10^{-07}	ZNF608	zinc_finger_protein_608
2.914	15.619	-2.419	3.25 x10^{-07}	BAALC	brain_and_acute_leukemia_cytoplasmic
48.377	219.550	-2.182	3.99 x10^{-07}	TNFSF10	tumor_necrosis_factor_(ligand)_superfamily_member_10

136.600	614.022	-2.168	4.25 $\times 10^{-07}$	MX2	myxovirus_(influenza_virus)_resistance_2_(mouse)
0.211	2.884	-3.724	4.69 $\times 10^{-07}$	NDC80	NDC80_kinetochore_complex_component
38.20	170.894	-2.161	5.48 $\times 10^{-07}$	PARP10	poly_(ADP-ribose)_polymerase_family_member_10
0.158	2.503	-3.921	7.30 $\times 10^{-07}$	LRRC3DN	LRRC3_downstream_neighbor_(non-protein_coding)
2.649	13.660	-2.363	7.51 $\times 10^{-07}$	DLGAP1-AS2	DLGAP1_antisense_RNA_2
1.854	10.123	-2.444	7.73 $\times 10^{-07}$	STAP1	signal_transducing_adaptor_family_member_1
1.165	7.238	-2.627	7.82 $\times 10^{-07}$	SLC1A2	solute_carrier_family_1_(glial_high_affinity_glutamate_transporter)_member_2
0.476	4.081	-3.079	8.04 $\times 10^{-07}$	MUC1	mucin_1_cell_surface_associated
8.107	37.172	-2.196	8.35 $\times 10^{-07}$	AK4	adenylate_kinase_4
0.105	2.231	-4.311	8.61 $\times 10^{-07}$	IL36RN	interleukin_36_receptor_antagonist
2.755	0.217	3.621	9.87 $\times 10^{-07}$	TIGD3	tigger_transposable_element_derived_3
3.179	0.326	3.257	1.46 $\times 10^{-06}$	HIST1H2AG	histone_cluster_1_H2ag
0.317	3.156	-3.284	1.46 $\times 10^{-06}$	GAPDHP14	glyceraldehyde-3-phosphate_dehydrogenase_pseudogene_14
237.328	983.024	-2.050	1.54 $\times 10^{-06}$	OAS2	2'-5'-oligoadenylate_synthetase_2_69/71kDa
37.196	155.70	-2.065	1.61 $\times 10^{-06}$	PRF1	perforin_1_(pore_forming_protein)
89.760	369.328	-2.040	1.80 $\times 10^{-06}$	XAF1	XIAP_associated_factor_1
0.529	4.08187	-2.929	1.82 $\times 10^{-06}$	XCR1	chemokine_(C_motif)_receptor_1
47.953	198.433	-2.048	1.82 $\times 10^{-06}$	SP110	SP110_nuclear_body_protein
1.006	6.095	-2.590	2.25 $\times 10^{-06}$	ASB2	ankyrin_repeat_and_SOCS_box_containing_2
0.423	3.537	-3.041	2.27 $\times 10^{-06}$	PRR16	proline_rich_16
4.821	21.606	-2.162	2.29 $\times 10^{-06}$	DAGLA	diacylglycerol_lipase_alpha
72.274	292.969	-2.019	2.31 $\times 10^{-06}$	EPSTI1	epithelial_stromal_interaction_1_(breast)
11.551	2.340	2.3	2.37 $\times 10^{-06}$	HPN	Hepsin
0	1.415	-7.726	2.40 $\times 10^{-06}$	PDZD2	PDZ_domain_containing_2
19.022	78.099	-2.037	2.63 $\times 10^{-06}$	TNFSF13B	tumor_necrosis_factor_(ligand)_superfamily_member_13b
0.688	4.626	-2.735	2.94 $\times 10^{-06}$	VNN3	vanin_3
4.821	0.761	2.651	3.02 $\times 10^{-06}$	MUC20	mucin_20_cell_surface_associated
2.755	0.272	3.308	3.22 $\times 10^{-06}$	FRY-AS1	FRY_antisense_RNA_1
1.483	7.837	-2.395	3.40 $\times 10^{-06}$	CA13	carbonic_anhydrase_XIII
10.756	2.231	2.265	3.88 $\times 10^{-06}$	RASL11A	RAS-like_family_11_member_A
33.699	8.218	2.035	4.47 $\times 10^{-06}$	ARRB1	arrestin_beta_1
15.048	59.540	-1.983	5.09 $\times 10^{-06}$	MB21D1	Mab-21_domain_containing_1
25.221	6.095	2.047	5.61 $\times 10^{-06}$	NRARP	NOTCH-regulated_ankyrin_repeat_protein
20.664	80.385	-1.959	5.85 $\times 10^{-06}$	IPCEF1	interaction_protein_for_cytohesin_exchange_factors_1
15.207	59.486	-1.967	6.01 $\times 10^{-06}$	ZBP1	Z-DNA_binding_protein_1
140.044	529.609	-1.919	6.39 $\times 10^{-06}$	IFIH1	interferon_induced_with_helicase_C_domain_1
16.531	64.003	-1.952	6.86 $\times 10^{-06}$	AHRR	aryl-hydrocarbon_receptor_repressor
95.111	356.266	-1.905	7.53 $\times 10^{-06}$	PML	promyelocytic_leukemia
68.830	257.702	-1.904	7.78 $\times 10^{-06}$	APOBEC3A	apolipoprotein_B_mRNA_editing_enzyme_catalytic_polypeptide-like_3A
0	1.251	-7.550	8.28 $\times 10^{-06}$	DFNB59	deafness_autosomal_recessive_59
0.900	5.115	-2.496	8.34 $\times 10^{-06}$	IL1RL2	interleukin_1_receptor-like_2

2.702	11.755	-2.118	$8.57\ x10^{-06}$	XIRP1	xin_actin-binding_repeat_containing_1
159.543	588.279	-1.882	$9.26\ x10^{-06}$	IFIT1	interferon-induced_protein_with_tetratricopeptide_repeats_1
0.370	2.938	-2.963	$9.35\ x10^{-06}$	CCL18	chemokine_(C-motif)_ligand_18_(pulmonary_and_activation-regulated)
4.397	0.761	2.518	$1.04\ x10^{-05}$	CD27-AS1	CD27_antisense_RNA_1
386.063	1410.47	-1.869	$1.04\ x10^{-05}$	ISG15	ISG15_ubiquitin-like_modifier
16.002	60.248	-1.912	$1.05\ x10^{-05}$	ADORA2A-AS1	ADORA2A_antisense_RNA_1
1.642	7.782	-2.239	$1.13\ x10^{-05}$	C1QTNF1	C1q_and_tumor_necrosis_factor_related_protein_1
84.037	22.695	1.888	$1.18\ x10^{-05}$	IL18	interleukin_18_(interferon-gamma-inducing_factor)
2.172	0.217	3.279	$1.38\ x10^{-05}$	CLEC12B	C-type_lectin_domain_family_12_member_B
0.741	4.299	-2.524	$1.42\ x10^{-05}$	PRKAG2-AS1	PRKAG2_antisense_RNA_1
23.579	85.937	-1.865	$1.45\ x10^{-05}$	APOL1	apolipoprotein_L_1
125.897	450.203	-1.838	$1.47\ x10^{-05}$	TRIM22	tripartite_motif_containing_22
69.783	19.103	1.868	$1.51\ x10^{-05}$	CPM	carboxypeptidase_M
183.441	51.213	1.840	$1.54\ x10^{-05}$	GADD45A	growth_arrest_and_DNA-damage-inducible_alpha
1575.99	443.509	1.829	$1.56\ x10^{-05}$	NEAT1	nuclear_paraspeckle_assembly_transcript_1_(non-protein_coding)
3.285	13.388	-2.0247	$1.57\ x10^{-05}$	LAG3	lymphocyte-activation_gene_3
0.052	1.469	-4.628	$1.58\ x10^{-05}$	PCDH17	protocadherin_17

B.

Symbol	Entrez Gene Name	Res	LPS	LPS + Res
CSF3	colony stimulating factor 3	4.059	14.018	15.135
RHCG	Rh family C glycoprotein		11.507	11.372
CLEC12A-AS1	CLEC12A antisense RNA 1	4.059	11.252	10.482
FRMD7	FERM domain containing 7		10.739	10.655
CFAP58-DT	CFAP58 divergent transcript	4.059	10.449	10.067
IL6	interleukin 6	-0.095	9.67	10.139
IL12B	interleukin 12B		9.513	8.337
RANBP3L	RAN binding protein 3 like		9.222	7.134
TMEM54	transmembrane protein 54		9.063	6.598
IL1A	interleukin 1 alpha	-1.366	8.788	8.899
ANO5	anoctamin 5	4.614	8.742	8.337
CCL20	C-C motif chemokine ligand 20	0.641	8.674	9.215
KCNJ2-AS1	KCNJ2 antisense RNA 1	2.13	8.637	8.949
IDO2	indoleamine 2,3-dioxygenase 2		8.616	6.836
HPN	hepsin	4.614	8.583	10.892
IFNB1	interferon beta 1		8.443	7.453
TSLP	thymic stromal lymphopoietin	5.015	8.405	8.216
MAS1	MAS1 proto-oncogene, G protein-coupled receptor	4.614	8.113	6.942
FGF13	fibroblast growth factor 13		8.065	5.478
MMP10	matrix metallopeptidase 10		8.065	8.449
NBEAP1	neurobeachin pseudogene 1	5.015	7.914	5.478

TNFSF18	TNF superfamily member 18		7.745	5.478
INHBA	inhibin subunit beta A	-0.476	7.711	5.3
NR5A2	nuclear receptor subfamily 5 group A member 2	5.015	7.684	7.22
ADCY10	adenylate cyclase 10	5.803	7.684	7.774
F3	coagulation factor III, tissue factor	-0.819	7.579	6.457
C2CD4B	C2 calcium dependent domain containing 4B	5.015	7.553	10.428
ACOD1	aconitate decarboxylase 1	0.789	7.546	8.656
MAFF	nuclear receptor binding factor 2 pseudogene 2		7.483	5.478
TCP10/TCP10L2	t-complex 10		7.483	5.954
ADAD2	adenosine deaminase domain containing 2	3.143	7.483	6.462
IGHV1-3	immunoglobulin heavy variable 1-3	5.328	7.483	6.462
MIR3945HG	MIR3945 host gene	-1.143	7.453	5.923
ABCA13	ATP binding cassette subfamily A member 13	3.143	7.41	5.736
XIAPP3	X-linked inhibitor of apoptosis pseudogene 3		7.41	8.036
TMEM26	transmembrane protein 26		7.332	5.164
BHLHE22	basic helix-loop-helix family member e22	3.143	7.332	
IL1B	interleukin 1 beta	-0.956	7.304	7.467
ACAN	aggrecan	4.059	7.25	6.942
HSPE1P4	heat shock protein family E (Hsp10) member 1 pseudogene 4		7.163	5.954
PTGS2, COX2	prostaglandin-endoperoxide synthase 2	-0.541	7.018	7.396
SERPINB7	serpin family B member 7		6.972	5.478
TEX41	testis expressed 41		6.972	5.478
RPL23AP93	ribosomal protein L23a pseudogene 93		6.972	6.722
DNAAF1	dynein axonemal assembly factor 1	0.045	6.93	5.969
HSD11B1	hydroxysteroid 11-beta dehydrogenase 1		6.866	6.311
TMEM139	transmembrane protein 139	5.328	6.752	5.164
CKMT2	creatine kinase, mitochondrial 2	4.614	6.752	5.478
CCDC116	coiled-coil domain containing 116	5.992	6.752	6.942
TNFAIP6	TNF alpha induced protein 6	0.801	6.695	7.742

C.

Symbol	Entrez Gene Name	Res	LPS	LPS + Res
DMBX1	diencephalon/mesencephalon homeobox 1	-5.645	-5.479	-5.504
LINC01013	long intergenic non-protein coding RNA 1013	-5.645	-5.479	-5.504
ZNRF2P1	zinc and ring finger 2 pseudogene 1	0.514	-5.479	-5.504
UTS2	urotensin 2	-1.587	-5.479	-5.504
TNFRSF17	TNF receptor superfamily member 17	-2.503	-5.479	-5.504
BRCC3P1	BRCA1/BRCA2-containing complex subunit 3 pseudogene 1	-0.631	-5.479	-5.504
TRAJ14	T cell receptor alpha joining 14	-0.061	-5.479	-5.504
TMC3-AS1	TMC3 antisense RNA 1	-2.503	-5.479	-5.504
LINC01629	long intergenic non-protein coding RNA 1629	0.197	-5.223	-5.247
GDF6	growth differentiation factor 6	-5.388	-5.223	-5.247
CA8	carbonic anhydrase 8	-5.388	-5.223	-5.247
ELOVL3	ELOVL fatty acid elongase 3	-0.774	-5.223	-5.247
DKFZp779M0652	uncharacterized DKFZp779M0652	-0.373	-5.223	-5.247
CD163L1	CD163 molecule like 1	0.415	-5.223	-5.247
KLC3	kinesin light chain 3	0.604	-5.223	-5.247
UVRAG-DT	UVRAG divergent transcript	-5.388	-5.223	-5.247

IQCJ-SCHIP1-AS1	IQCJ-SCHIP1 read through antisense RNA 1	-5.388	-5.223	-5.247
LINC00520	long intergenic non-protein coding RNA 520	-5.388	-5.223	-5.247
DNMT3L	DNA methyltransferase 3 like	-5.388	-5.223	-5.247
PRRG2	proline rich and Gla domain 2	0.415	-5.223	-5.247
GRM4	glutamate metabotropic receptor 4	-5.388	-5.223	-5.247
METTL24	methyltransferase like 24	-5.388	-5.223	-5.247
RPL26P27	ribosomal protein L26 pseudogene 27	0.604	-5.223	-5.247
PGM5	phosphoglucomutase 5	-0.061	-5.479	-2.224
ANKRD33	ankyrin repeat domain 33	-1.031	-5.479	-2.224
RBP4	retinol binding protein 4	-1.587	-5.479	-2.224
TEX21P	testis expressed 21, pseudogene	-0.318	-5.479	-2.224

*These data represent the genes that are downregulated by LPS and not modulated (genes downregulated/upregulated) by resveratrol, except for the last four, in PBMCs (incubated for 3h). RNAs were extracted and subjected to RNAseq analysis. This data has been obtained by using the DEG program.

**These data represent the genes that are not greatly modulated (genes downregulated/upregulated) by resveratrol + LPS as compared to LPS alone in PBMCs (incubated for 3h). RNAs were extracted and subjected to RNAseq analysis. This data has been obtained by using the DEG program. The log2 ratios were imported into the Ingenuity Pathways Analysis. These numbers represent the z scores for the genes.

TABLE S3. (A) GENES MODULATED BY LEC10 ALONE*. (B) GENES NOT UPREGULATED BY LEC10 TO THE SAME EXTENT AS LPS*. (C) GENES THAT ARE UPREGULATED BY LPS, LEC10, LEC10 + LPS, LEC50 AND LEC50 + LPS. (D) GENES DOWNREGULATED BY LPS AND LECTINS**.**

A.

LEC10	control1	Log2 Fold	*P* value	Gene Name	Gene Description
1105.12	2.180	8.980	1.96 $\times 10^{-51}$	IL6	interleukin_6_(interferon_beta_2)
1116.645	2.824	8.623	2.62 $\times 10^{-49}$	IL1A	interleukin_1_alpha
2159.807	7.532	8.162	3.34 $\times 10^{-47}$	CCL4	chemokine_(C-C_motif)_ligand_4
226.943	0.446	8.969	5.46 $\times 10^{-45}$	CCL3L1	chemokine_(C-C_motif)_ligand_3-like_1
77.794	0	13.49	1.22 $\times 10^{-40}$	CSF3	colony_stimulating_factor_3_(granulocyte)
19385.16	146.59	7.046	8.24 $\times 10^{-40}$	IL1B	interleukin_1_beta
199.06	0.941	7.713	2.26 $\times 10^{-39}$	CCL20	chemokine_(C-C_motif)_ligand_20
488.089	3.270	7.218	3.70 $\times 10^{-39}$	F3	coagulation_factor_III_(thromboplastin_tissue_factor)
2886.299	31.916	6.498	1.50 $\times 10^{-35}$	PTGS2, COX2	Prostaglandin-endoperoxide_synthase_2_(prostaglandin_G/H_synthase_and_cyclooxygenase)
178.034	1.784	6.635	1.10 $\times 10^{-33}$	IRG1	immunoresponsive_1_homolog_(mouse)
128.475	1.139	6.808	2.52 $\times 10^{-33}$	CCL3	chemokine_(C-C_motif)_ligand_3
120.973	1.139	6.721	1.07 $\times 10^{-32}$	KCNJ2	potassium_inwardly-rectifying_channel_subfamily_J_member_2
240.234	2.973	6.332	1.47 $\times 10^{-32}$	INHBA	inhibin_beta_A
546.862	7.681	6.152	2.16 $\times 10^{-32}$	TNFAIP6	tumor_necrosis_factor_alpha-induced_protein_6
692.586	10.159	6.090	4.05 $\times 10^{-32}$	CXCL1	chemokine_(C-X-C_motif)_ligand_1 (melanoma_growth_stimulating_activity_alpha)
30.479	0.049	9.080	7.20 $\times 10^{-31}$	CCL4L2	chemokine_(C-C_motif)_ligand_4-like_2
20.792	0.049	8.529	5.16 $\times 10^{-27}$	KCNJ2-AS1	KCNJ2_antisense_RNA_1_(head_to_head)
43.770	0.644	6.071	4.23 $\times 10^{-26}$	DNAAF1	dynein_axonemal_assembly_factor_1

54.343	1.288	5.391	7.87 x10^{-24}	TNIP3	TNFAIP3_interacting_protein_3
26.226	0.346	6.212	9.88 x10^{-24}	CSF2	colony_stimulating_factor_2_(granulocyte-macrophage)
439.770	15.065	4.866	1.58 x10^{-23}	TNF	tumor_necrosis_factor
2759.83	109.77	4.651	1.99 x10^{-22}	CXCL2	chemokine_(C-X-C_motif)_ligand_2
2523.37	105.21	4.583	5.92 x10^{-22}	CXCL3	chemokine_(C-X-C_motif)_ligand_3
50.917	1.932	4.714	3.29 x10^{-20}	ADORA2A	adenosine_A2a_receptor
32.960	1.189	4.784	1.64 x10^{-19}	IL7	interleukin_7
12.109	0.148	6.284	1.99 x10^{-19}	C1QTNF1	C1q_and_tumor_necrosis_factor_related_protein_1
40.462	1.685	4.580	7.24 x10^{-19}	ADORA2A-AS1	ADORA2A_antisense_RNA_1
278.038	18.584	3.902	4.97 x10^{-17}	GJB2	gap_junction_protein_beta_2_26kDa
435.162	30.825	3.819	1.31 x10^{-16}	RIN2	Ras_and_Rab_interactor_2
14.471	0.594	4.589	6.57 x10^{-16}	SYNPO2	synaptopodin_2
6.202	0	9.848	6.77 x10^{-16}	CCDC147-AS1	CCDC147_antisense_RNA_1_(head_to_head)
6.143	0	9.834	7.99 x10^{-16}	FRMD7	FERM_domain_containing_7
29.062	1.685	4.102	9.20 x10^{-16}	UPB1	ureidopropionase_beta
7.974	0.148	5.682	1.10 x10^{-15}	IL21-AS1	IL21_antisense_RNA_1
13.999	0.594	4.541	1.23 x10^{-15}	ZP3	zona_pellucida_glycoprotein_3_(sperm_receptor)
16.657	0.792	4.381	1.58 x10^{-15}	FLT1	fms-related_tyrosine_kinase_1
3340.246	272.525	3.615	1.85 x10^{-15}	SERPINB2	serpin_peptidase_inhibitor_clade_B_(ovalbumin)_member_2
38.572	2.626	3.872	3.03 x10^{-15}	PDSS1	prenyl_(decaprenyl)_diphosphate_synthase_subunit1
8.210	0.198	5.325	4.05 x10^{-15}	IL36G	interleukin_36_gamma
30181.18	2646.306	3.511	8.38 x10^{-15}	IL8	interleukin_8
8.801	0.297	4.856	2.69 x10^{-14}	CASP5	caspase_5_apoptosis-related_cysteine_peptidase
3196.295	297.998	3.423	3.11 x10^{-14}	EREG	epiregulin
20.142	1.338	3.905	3.99 x10^{-14}	FFAR2	free_fatty_acid_receptor_2
21.442	1.486	3.844	6.12 x10^{-14}	PLD1	phospholipase_D1_phosphatidylcholine-specific
4.489	50.897	-3.501	1.72 x10^{-13}	DHRS9	dehydrogenase/reductase_(SDR_family)_member_9
167.165	16.651	3.327	2.48 x10^{-13}	IFIT1	interferon-induced_protein_with_tetratricopeptide_repeats_1
494.409	51.244	3.270	3.12 x10^{-13}	BCL2A1	BCL2-related_protein_A1
4.252	0	9.304	6.26 x10^{-13}	RHCG	Rh_family_C_glycoprotein
1013.096	109.723	3.206	7.13 x10^{-13}	NFKBIZ	nuclear_factor_of_kappa_light_polypeptide_gene_enhancer_in_B-cells_inhibitor_zeta
44.715	4.311	3.372	8.39 x10^{-13}	CD274	CD274_molecule
9.982	0.594	4.053	9.45 x10^{-13}	C1orf61	chromosome_1_open_reading_frame_61
1006.126	111.260	3.176	1.09 x10^{-12}	IFIT3	interferon-induced_protein_with_tetratricopeptide_repeats_3
62.495	6.343	3.299	1.16 x10^{-12}	hsa-mir-146a	hsa-mir-146a
659.862	74.437	3.147	1.68 x10^{-12}	G0S2	G0/G1switch_2
758.803	87.323	3.119	2.47 x10^{-12}	NLRP3	NLR_family_pyrin_domain_containing_3
4.607	0.049	6.356	2.91 x10^{-12}	ZNF259P1	zinc_finger_protein_259_pseudogene_1

6.852	0.297	4.495	2.94 x10^{-12}	SLC9A7P1	solute_carrier_family_9_subfamily_A_(NHE7_cation_proton_antiporter_7)_member_7_pseudogene_1
107.269	11.943	3.166	3.06 x10^{-12}	ITGB8	integrin_beta_8
1728.247	203.043	3.089	3.57 x10^{-12}	ACSL1	acyl-CoA_synthetase_long-chain_family_member_1
122.982	14.173	3.116	5.18 x10^{-12}	MIR155HG	MIR155_host_gene_(non-protein_coding)
14.176	1.139	3.628	6.36 x10^{-12}	GBP1P1	guanylate_binding_protein_1_interferon-inducible_pseudogene_1
10.278	0.743	3.777	8.84 x10^{-12}	PNPLA1	patatin-like_phospholipase_domain_containing_1
14.5901	1.288	3.494	1.18 x10^{-11}	STEAP4	STEAP_family_member_4
9.155729	0.644	3.815	1.33 x10^{-11}	CDK1	cyclin-dependent_kinase_1
147.1414	18.634	2.980	2.81 x10^{-11}	KANK1	KN_motif_and_ankyrin_repeat_domains_1
156.179	19.873	2.973	2.99 x10^{-11}	CLEC4E	C-type_lectin_domain_family_4_member_E
3.957	0.049	6.137	3.78 x10^{-11}	CCL4L1	chemokine_(C-C_motif)_ligand_4-like_1
170.946	22.450	2.928	5.01 x10^{-11}	CMPK2	cytidine_monophosphate_(UMP-CMP)_kinase_2_mitochondrial
16.716	1.685	3.305	5.49 x10^{-11}	SCN1B	sodium_channel_voltage-gated_type_I_beta_subunit
857.980	118.148	2.860	8.22 x10^{-11}	IFIT2	interferon-induced_protein_with_tetratricopeptide_repeats_2
5.198	0.247	4.353	9.54 x10^{-11}	ABCA6	ATP-binding_cassette_sub-family_A_(ABC1)_member_6
282.646	38.903	2.860	1.01 x10^{-10}	RNF144B	ring_finger_protein_144B
1107.666	154.574	2.841	1.05 x10^{-10}	IL1RN	interleukin_1_receptor_antagonist
0.945	10.803	-3.50	1.28 x10^{-10}	PDK4	pyruvate_dehydrogenase_kinase_isozyme_4
384.599	54.217	2.826	1.38 x10^{-10}	ISG15	ISG15_ubiquitin-like_modifier
53.752	7.186	2.901	1.83 x10^{-10}	MFSD2A	major_facilitator_superfamily_domain_containing_2A
14.471	1.536	3.230	1.86 x10^{-10}	ELOVL7	ELOVL_fatty_acid_elongase_7
560.389	80.979	2.790	2.12 x10^{-10}	ETS2	ets_erythroblastosis_virus_E26_oncogene_homolog_2_(avian)
96.696	13.480	2.842	2.25 x10^{-10}	CXCL10	chemokine_(C-X-C_motif)_ligand_10
170.355	24.531	2.795	2.84 x10^{-10}	CXCL5	chemokine_(C-X-C_motif)_ligand_5
314.543	46.089	2.770	3.18 x10^{-10}	DRAM1	DNA-damage_regulated_autophagy_modulator_1
126.821	18.535	2.774	4.29 x10^{-10}	XAF1	XIAP_associated_factor_1
7.442	0.594	3.630	4.91 x10^{-10}	CXCL11	chemokine_(C-X-C_motif)_ligand_11
965.663	151.997	2.667	1.01 x10^{-09}	SOCS3	suppressor_of_cytokine_signaling_3
7.501	0.693	3.421	1.02 x10^{-09}	E2F7	E2F_transcription_factor_7
907.126	144.316	2.652	1.23 x10^{-09}	SLC39A8	solute_carrier_family_39_(zinc_transporter)_member8
135.682	21.211	2.676	1.36 x10^{-09}	IFIT5	interferon-induced_protein_with_tetratricopeptide_repeats_5
40.462	5.996	2.753	1.59 x10^{-09}	PDGFB	platelet-derived_growth_factor_beta_polypeptide
4.312	0.247	4.084	1.91 x10^{-09}	INHBA-AS1	INHBA_antisense_RNA_1
3.485	0.148	4.489	1.93 x10^{-09}	OR2B11	olfactory_receptor_family_2_subfamily_B_member_11
505.455	82.714	2.611	2.15 x10^{-09}	SLAMF7	SLAM_family_member_7
5.670	0.446	3.648	2.85 x10^{-09}	EDN1	endothelin_1

31.602	4.807	2.715	3.62 x10^{-09}	WNT5A	wingless-type_MMTV_integration_site_family_member_5A
12225.97	2086.338	2.550	4.23 x10^{-09}	SOD2	superoxide_dismutase_2_mitochondrial
492.991	84.002	2.553	4.47 x10^{-09}	GCH1	GTP_cyclohydrolase_1
65.035	10.655	2.608	4.95 x10^{-09}	AK4	adenylate_kinase_4
19.256	2.775	2.791	5.42 x10^{-09}	MRPS24	mitochondrial_ribosomal_protein_S24
1571.064	274.210	2.518	6.49 x10^{-09}	PLAUR	plasminogen_activator_urokinase_receptor
11.636	69.779	-2.58	7.17 x10^{-09}	C5AR2	complement_component_5a_receptor_2
22.210	3.370	2.717	7.90 x10^{-09}	LAMP3	lysosomal-associated_membrane_protein_3
4.961	0.396	3.623	8.37 x10^{-09}	LINC00346	long_intergenic_non-protein_coding_RNA_346
14.058	1.982	2.821	9.35 x10^{-09}	CD80	CD80_molecule
2.244	0	8.384	1.09 x10^{-08}	IL12B	interleukin_12B_(natural_killer_cell_stimulatory_factor_2_cytotoxic_lymphocyte_maturation_factor_2_p40)
70.705	12.191	2.535	1.15 x10^{-08}	OAS1	2'-5'-oligoadenylate_synthetase_1_40/46kDa
54.107	298.296	-2.46	1.45 x10^{-08}	CEBPD	CCAAT/enhancer_binding_protein_(C/EBP)_delta
216.784	39.300	2.463	1.60 x10^{-08}	HS3ST3B1	heparan_sulfate_(glucosamine)_3-O-sulfotransferase_3B1
4.371	0.346	3.629	2.01 x10^{-08}	EHF	ets_homologous_factor
2.126	0	8.306	2.10 x10^{-08}	IDO2	indoleamine_23-dioxygenase_2
52.926	284.072	-2.42	2.33 x10^{-08}	HMOX1	heme_oxygenase_(decycling)_1
6.674	38.556	-2.52	2.50 x10^{-08}	SERPINE1	serpin_peptidase_inhibitor_clade_E_(nexin_plasminogen_activator_inhibitor_type_1)_member_1
11.164	1.585	2.810	2.61 x10^{-08}	AP003733.1	Uncharacterized_protein;_cDNA_FLJ36460_fis_clone_THYMU2014801_
563.106	106.254	2.405	2.68 x10^{-08}	SLC2A6	solute_carrier_family_2_(facilitated_glucose_transporter)_member_6
7.324	0.892	3.028	2.71 x10^{-08}	HS3ST3A1	heparan_sulfate_(glucosamine)_3-O-sulfotransferase_3A1
4.489	26.761	-2.57	3.01 x10^{-08}	SDS	serine_dehydratase
416.615	79.344	2.392	3.22 x10^{-08}	GBP1	guanylate_binding_protein_1_interferon-inducible
3.248	0.198	3.989	3.42 x10^{-08}	GCKR	glucokinase_(hexokinase_4)_regulator
13.113	2.031	2.686	4.17 x10^{-08}	C11orf96	chromosome_11_open_reading_frame_96
191.325	36.921	2.373	4.81 x10^{-08}	OAS3	2'-5'-oligoadenylate_synthetase_3_100kDa
6.852	37.912	-2.46	4.85 x10^{-08}	CDH6	cadherin_6_type_2_K-cadherin_(fetal_kidney)
63.558	12.042	2.399	5.77 x10^{-08}	TRIP10	thyroid_hormone_receptor_interactor_10
317.851	62.543	2.345	5.78 x10^{-08}	MX1	myxovirus_(influenza_virus)_resistance_1_interferon-inducible_protein_p78_(mouse)
1413.054	279.711	2.336	5.88 x10^{-08}	IER3	immediate_early_response_3
9.746	1.437	2.755	6.74 x10^{-08}	C12orf61	chromosome_12_open_reading_frame_61
350.989	70.274	2.320	7.69 x10^{-08}	CLIC4	chloride_intracellular_channel_4
15.062	2.527	2.572	7.81 x10^{-08}	TEX14	testis_expressed_14
3.662	0.297	3.592	8.40 x10^{-08}	AL135998.	Uncharacterized_protein_
94.215	18.683	2.333	9.70 x10^{-08}	ADCY9	adenylate_cyclase_9
3.603	0.297	3.569	1.04 x10^{-07}	CARD17	caspase_recruitment_domain_family_member_17
254.411	51.987	2.290	1.12 x10^{-07}	MMP14	matrix_metallopeptidase_14_(membrane-inserted)

48.850	9.564	2.351	1.18 x10^{-07}	IL15RA	interleukin_15_receptor_alpha
449.457	92.625	2.278	1.22 x10^{-07}	HELZ2	helicase_with_zinc_finger_2_transcriptional_coactivator
1101.818	228.021	2.272	1.26 x10^{-07}	TRAF1	TNF_receptor-associated_factor_1
602.092	125.037	2.267	1.37 x10^{-07}	ICAM1	intercellular_adhesion_molecule_1
66.275	13.380	2.307	1.59 x10^{-07}	KMO	kynurenine_3-monooxygenase_(kynurenine_3-hydroxylase)
35.264	170.185	-2.270	1.63 x10^{-07}	FUCA1	fucosidase_alpha-L-_1_tissue
41.880	8.325	2.329	1.72 x10^{-07}	SFR1	SWI5-dependent_recombination_repair_1
192.624	40.192	2.260	1.74 x10^{-07}	CCRL2	chemokine_(C-C_motif)_receptor-like_2
4.193	22.252	-2.40	2.10 x10^{-07}	IFI30	interferon_gamma-inducible_protein_30
8.269	1.288	2.675	2.13 x10^{-07}	MGAM	maltase-glucoamylase_(alpha-glucosidase)
294.991	63.683	2.211	2.75 x10^{-07}	RSAD2	radical_S-adenosyl_methionine_domain_containing_2
3.603	0.346	3.351	3.24 x10^{-07}	PPP1R17	protein_phosphatase_1_regulatory_subunit_17
1.713	0	7.995	3.48 x10^{-07}	FGF13	fibroblast_growth_factor_13
212.176	46.337	2.194	3.61 x10^{-07}	ID2	inhibitor_of_DNA_binding_2_dominant_negative_helix-loop-helix_protein
1.594	9.664	-2.59	3.68 x10^{-07}	NLRC4	NLR_family_CARD_domain_containing_4
6.674	0.991	2.743	3.83 x10^{-07}	CHAC1	ChaC_cation_transport_regulator_homolog_1_(E._coli)
173.427	37.962	2.191	3.89 x10^{-07}	ARL5B	ADP-ribosylation_factor-like_5B
2.008	0.049	5.161	4.78 x10^{-07}	TNFSF15	tumor_necrosis_factor_(ligand)_superfamily_member_15
330.492	74.090	2.157	5.02 x10^{-07}	OAS2	2'-5'-oligoadenylate_synthetase_2_69/71kDa
102.485	22.747	2.171	5.71 x10^{-07}	SAV1	salvador_homolog_1_(Drosophila)
4.016	20.071	-2.31	6.12 x10^{-07}	A4GALT	alpha_14-galactosyltransferase
2.303	0.148	3.894	6.40 x10^{-07}	CCL23	chemokine_(C-C_motif)_ligand_23
950.246	218.605	2.119	7.18 x10^{-07}	PELI1	pellino_E3_ubiquitin_protein_ligase_1
1.594	0	7.893	7.40 x10^{-07}	LINC00158	long_intergenic_non-protein_coding_RNA_158
1.594	0	7.893	7.40 x10^{-07}	DAPK1-IT1	DAPK1_intronic_transcript_1_(non-protein_coding)
20.910	4.361	2.259	8.42 x10^{-07}	BRE	brain_and_reproductive_organ-expressed_(TNFRSF1A_modulator)
6.556	1.040	2.647	8.45 x10^{-07}	IL19	interleukin_19
0.945	6.194	-2.70	1.02 x10^{-06}	GPR162	G_protein-coupled_receptor_162
2.185	0.148	3.818	1.16 x10^{-06}	LINC00515	long_intergenic_non-protein_coding_RNA_515
13.231	2.676	2.302	1.38 x10^{-06}	TLR7	toll-like_receptor_7
0.295	3.171	-3.39	1.46 x10^{-06}	PVRL4	poliovirus_receptor-related_4
1.476	0	7.782	1.61 x10^{-06}	C2CD4B	C2_calcium-dependent_domain_containing_4B
4.430	0.594	2.883	1.64 x10^{-06}	BCL2L14	BCL2-like_14_(apoptosis_facilitator)
0.708	5.055	-2.822	1.66 x10^{-06}	PTGFRN	prostaglandin_F2_receptor_inhibitor
1.831	0.049	5.028	1.83 x10^{-06}	IL12A	interleukin_12A_(natural_killer_cell_stimulatory_factor_1_cytotoxic_lymphocyte_maturation_factor_1_p35)
6.852	1.238	2.461	2.12 x10^{-06}	FAR2	fatty_acyl_CoA_reductase_2

123.100	30.082	2.032	2.35×10^{-06}	ZC3H12C	zinc_finger_CCCH-type_containing_12C
4.961	0.792	2.635	2.60×10^{-06}	PPP4R4	protein_phosphatase_4_regulatory_subunit_4
1716.965	432.254	1.989	2.93×10^{-06}	EHD1	EH-domain_containing_1
1456.352	367.331	1.987	3.02×10^{-06}	CD83	CD83_molecule
8.210	1.585	2.367	3.09×10^{-06}	ZEB2-AS1	ZEB2_antisense_RNA_1
38.808	9.317	2.057	3.21×10^{-06}	SGPP2	sphingosine-1-phosphate_phosphatase_2
238.462	60.164	1.986	3.32×10^{-06}	MYC	v-myc_myelocytomatosis_viral_oncogene_homolog_(avian)
34.437	8.276	2.056	3.37×10^{-06}	HCAR2	hydroxycarboxylic_acid_receptor_2
1.358	0	7.662	3.61×10^{-06}	PCNPP3	PEST_containing_nuclear_protein_pseudogene_3
8130.583	2083.563	1.964	3.80×10^{-06}	THBS1	thrombospondin_1
3.248	0.396	3.013	3.90×10^{-06}	CAMK1G	calcium/calmodulin-dependent_protein_kinase_IG
925.673	237.784	1.960	4.04×10^{-06}	AQP9	aquaporin_9
5.670	0.991	2.508	4.20×10^{-06}	FERMT2	fermitin_family_member_2
388.143	100.059	1.955	4.42×10^{-06}	MAFF	vmaf_musculoaponeurotic_fibrosarcoma_oncogene_homolog_F_(avian)
2.480	0.247	3.288	4.66×10^{-06}	LINC00299	long_intergenic_non-protein_coding_RNA_299
1279.026	335.316	1.931	5.46×10^{-06}	TNFAIP2	tumor_necrosis_factor_alpha-induced_protein_2
9.510	2.031	2.222	5.92×10^{-06}	CMKLR1	chemokine-like_receptor_1
2.421	0.247	3.254	6.09×10^{-06}	WNT5B	wingless-type_MMTV_integration_site_family_member_5B
6.320	25.770	-2.02	6.42×10^{-06}	PFKFB4	6-phosphofructo-2-kinase/fructose-26-biphosphatase_4
3.603	15.363	-2.09	7.46×10^{-06}	LINGO3	leucine_rich_repeat_and_Ig_domain_containing_3
17.130	4.113	2.056	8.44×10^{-06}	HDAC9	histone_deacetylase_9
8.033	1.685	2.248	8.51×10^{-06}	GGT5	gamma-glutamyltransferase_5
134.087	35.930	1.899	9.23×10^{-06}	ADM	adrenomedullin
388.143	105.808	1.875	1.02×10^{-05}	PMAIP1	phorbol-12-myristate-13-acetate-induced_protein_1
11.459	43.760	-1.93	1.03×10^{-05}	CD300LB	CD300_molecule-like_family_member_b
11.282	43.165	-1.93	1.03×10^{-05}	TMEM170B	transmembrane_protein_170B
1.831	0.148	3.563	1.06×10^{-05}	COL17A1	collagen_type_XVII_alpha_1
63.794	17.147	1.895	1.18×10^{-05}	PSTPIP2	proline-serine-threonine_phosphatase_interacting_protein_2
27.526	7.136	1.946	1.18×10^{-05}	SMCO4	single-pass_membrane_protein_with_coiled-coil_domains_4
15.712	3.865	2.021	1.21×10^{-05}	ADAMDEC1	ADAM-like_decysin_1
1000.455	276.688	1.854	1.22×10^{-05}	PLEK	pleckstrin
407.400	112.697	1.853	1.26×10^{-05}	MSC	musculin
71.828	19.476	1.882	1.27×10^{-05}	SNX10	sorting_nexin_10
15.948	3.964	2.006	1.41×10^{-05}	RBKS	ribokinase

3.130	0.446	2.792	1.44 x10^{-05}	MAFG-AS1	MAFG_antisense_RNA_1_(head_to_head)
216.075	60.313	1.840	1.51 x10^{-05}	MX2	myxovirus_(influenza_virus)_resistance_2_(mouse)
249.094	69.630	1.838	1.52 x10^{-05}	EIF1B	eukaryotic_translation_initiation_factor_1B
4.725	0.892	2.396	1.53 x10^{-05}	SLC1A2	solute_carrier_family_1_(glial_high_affinity_glutamate_transporter)_member_2
1.476	0.049	4.719	1.58 x10^{-05}	ZDHHC4P1	zinc_finger_DHHC-type_containing_4_pseudogene_1
35.441	9.515	1.896	1.58 x10^{-05}	CDKN2B	cyclin-dependent_kinase_inhibitor_2B_(p15_inhibits_CDK4)
119.910	33.650	1.833	1.80 x10^{-05}	NBN	nibrin
1004.59	285.509	1.815	1.81 x10^{-05}	PNRC1	proline-rich_nuclear_receptor_coactivator_1
1.890	8.276	-2.12	1.85 x10^{-05}	NLRP12	NLR_family_pyrin_domain_containing_12
750.356	213.550	1.813	1.86 x10^{-05}	WTAP	Wilms_tumor_1_associated_protein
8.860	32.857	-1.89	1.86 x10^{-05}	LYL1	lymphoblastic_leukemia_derived_sequence_1
2.421	0.297	2.997	1.87 x10^{-05}	GPR141	G_protein-coupled_receptor_141
46.014	12.687	1.858	1.98 x10^{-05}	TMEM106	transmembrane_protein_106A
219.973	62.593	1.813	1.98 x10^{-05}	C15orf48	chromosome_15_open_reading_frame_48
13.054	47.428	-1.86	2.01 x10^{-05}	SLC37A2	solute_carrier_family_37_(glycerol-3-phosphate_transporter)_member_2
45.896	12.687	1.854	2.04 x10^{-05}	GJA3	gap_junction_protein_alpha_3_46kDa
842.740	241.749	1.801	2.08 x10^{-05}	MARCKS	myristoylated_alanine-rich_protein_kinase_C_substrate
28.648	7.7807	1.879	2.14 x10^{-05}	LRP12	low_density_lipoprotein_receptor-related_protein_12
16.952	4.410	1.940	2.16 x10^{-05}	SLC1A3	solute_carrier_family_1_(glial_high_affinity_glutamate transporter)_member_3
12.699	45.842	-1.85	2.17 x10^{-05}	PHF23	PHD_finger_protein_23
4.430	0.8425	2.385	2.17 x10^{-05}	PRG4	proteoglycan_4
3.839	0.693	2.456	2.18 x10^{-05}	ADRA2B	adrenoceptor_alpha_2B
24.513	86.877	-1.82	2.21 x10^{-05}	CXXC5	CXXC_finger_protein_5
10.573	38.259	-1.85	2.37 x10^{-05}	RAI14	retinoic_acid_induced_14
58.065	16.354	1.827	2.39 x10^{-05}	GADD45A	growth_arrest_and_DNA-damage-inducible_alpha
2673.886	779.316	1.778	2.56 x10^{-05}	NFKBIA	nuclear_factor_of_kappa_light_polypeptide_gene_enhancer_in_B-cells_inhibitor_alpha
26.226	7.186	1.866	2.60 x10^{-05}	HCAR3	hydroxycarboxylic_acid_receptor_3
4.607	17.593	-1.93	2.60 x10^{-05}	AVPI1	arginine_vasopressin-induced_1
134.323	38.755	1.793	2.65 x10^{-05}	PILRA	paired_immunoglobin-like_type_2_receptor_alpha
119.0245	34.493	1.786	2.87 x10^{-05}	SAMD9L	sterile_alpha_motif_domain_containing_9-like
1.653	0.099	3.971	2.88 x10^{-05}	BLACE	B-cell_acute_lymphoblastic_leukemia_expressed
1.653	0.148	3.417	2.88 x10^{-05}	CES1P1	carboxylesterase_1_pseudogene_1
30.361	8.474	1.840	2.96 x10^{-05}	DFNA5	deafness_autosomal_dominant_5
2.539	0.346	2.848	3.04 x10^{-05}	TULP2	tubby_like_protein_2
0	1.040	-7.28	3.05 x10^{-05}	PTPRH	protein_tyrosine_phosphatase_receptor_type_H

B.

LPS	LEC10	Log2 Fold	*P* value	Gene Name	Gene Description

66.397	6.369	3.380	3.99×10^{-13}	IFNG	Interferon-gamma
131.870	16.719	2.979	3.19×10^{-11}	USP18	ubiquitin_specific_peptidase_18
44.223	6.687	2.724	2.05×10^{-09}	CXCL11	chemokine_(C-X-C_motif)_ligand_11
1395.944	265.063	2.396	2.88×10^{-08}	RSAD2	radical_S-adenosyl_methionine_domain_containing_2
393.764	74.572	2.400	2.95×10^{-08}	IFI6	interferon_alpha-inducible_protein_6
15.275	2.653	2.521	1.50×10^{-07}	CACNA1A	calcium_channel_voltage-dependent_P/Q_type_alpha_1A_subunit
7.329	1.114	2.709	3.69×10^{-07}	FAM19A2	family_with_sequence_similarity_19_(chemokine_(C-C_motif)-like)_member_A2
91.465	19.956	2.196	4.74×10^{-07}	LAMP3	lysosomal-associated_membrane_protein_3
20.017	3.980	2.328	5.25×10^{-07}	BCL2L14	BCL2-like_14_(apoptosis_facilitator)
24.575	5.095	2.268	7.22×10^{-07}	SIGLEC1	sialic_acid_binding_Ig-like_lectin_1_sialoadhesin
1500.467	345.580	2.118	7.24×10^{-07}	ISG15	ISG15_ubiquitin-like_modifier
3.202	0.318	3.301	9.76×10^{-07}	CCNA1	cyclin_A1

C.

Symbol	Entrez Gene Name	LPS	LEC10	LEC10 + LPS	LEC50	LEC50 + LPS
IL6	interleukin 6	9.671	9.724	9.724	9.917	9.865
IL1A	interleukin 1 alpha	8.788	8.816	8.816	9.167	8.843
CCL20	C-C motif chemokine ligand 20	8.674	8.764	8.764	9.231	8.888
CSF3	colony stimulating factor 3	14.018	14.054	14.054	14.629	14.428
INHBA	inhibin subunit beta A	7.711	7.668	7.668	7.583	7.587
F3	coagulation factor III, tissue factor	7.579	7.674	7.674	8.117	7.857
IL1B	interleukin 1 beta	7.304	7.380	7.380	7.749	7.572
ACOD1	aconitate decarboxylase 1	7.546	7.539	7.539	7.252	7.811
MIR3945HG	MIR3945 host gene	7.453	7.541	7.541	7.489	7.526
PTGS2	prostaglandin-endoperoxide synthase 2	7.018	7.012	7.012	7.354	7.263
TNFAIP6	TNF alpha induced protein 6	6.695	6.671	6.671	6.757	6.659
DNAAF1	dynein axonemal assembly factor 1	6.930	7.036	7.036	6.981	7.141
CSF2	colony stimulating factor 2	7.311	7.399	7.399	8.025	7.84
CXCL11	C-X-C motif chemokine ligand 11	6.662	6.324	6.324	5.338	6.432
KCNJ2-AS1	KCNJ2 antisense RNA 1	8.637	8.511	8.511	8.857	8.535
RHCG	Rh family C glycoprotein	11.507	11.269	11.269	11.039	11.306
CLEC12A-AS1	CLEC12A antisense RNA 1	11.252	11.396	11.396	11.543	11.66
FRMD7	FERM domain containing 7	10.739	10.902	10.902	11.371	11.338
CFAP58-DT	CFAP58 divergent transcript	10.449	10.107	10.107	10.009	10.125
ZNF259P1	zinc finger protein 259 pseudogene 1	7.316	7.053	7.053	7.401	7.474
IL12B	interleukin 12B	9.513	9.761	9.761	9.497	9.525
RANBP3L	RAN binding protein 3 like	9.222	9.346	9.346	9.140	9.040
TMEM54	transmembrane protein 54	9.063	8.993	8.993	9.140	9.936
ANO5	anoctamin 5	8.742	8.449	8.449	7.394	8.663
IDO2	indoleamine 2,3-dioxygenase 2	8.616	9.145	9.145	8.351	9.084
HPN	hepsin	8.583	7.152	7.152	7.668	7.273
IL36RN	interleukin 36 receptor antagonist	8.515	7.942	7.942	8.142	8.774
LINC00158	long intergenic non-protein coding RNA 158	8.443	8.698	8.698	7.467	8.342
IFNB1	interferon beta 1	8.443	8.194	8.194	8.534	8.851

TSLP	thymic stromal lymphopoietin	8.405	8.369	8.369	8.998	8.574
PCNPP3	PEST containing nuclear protein pseudogene 3	8.245	8.731	8.731	8.312	8.511
MAS1	MAS1 proto-oncogene, G protein-coupled receptor	8.113	7.827	7.827	6.736	7.829
MMP10	matrix metallopeptidase 10	8.065	7.766	7.766	8.351	8.342
FGF13	fibroblast growth factor 13	8.065	8.597	8.597	9.094	9.392
LY6E-DT	LY6E divergent transcript	8.065	7.565	7.565	7.148	7.547
PCDH17	protocadherin 17	7.914	5.588	5.588	6.325	6.615
NBEAP1	neurobeachin pseudogene 1	7.914	6.709	6.709	7.234	7.777
PDGFRL	platelet derived growth factor receptor like	7.860	7.152	7.152	5.492	8.065
TNFSF18	TNF superfamily member 18	7.745	7.245	7.245	8.050	8.150
ADCY10	adenylate cyclase 10	7.684	7.414	7.414	6.612	6.491
NR5A2	nuclear receptor subfamily 5 group A member 2	7.684	6.709	6.709	7.668	7.777
C2CD4B	C2 calcium dependent domain containing 4B	7.553	7.766	7.766	8.39	8.065
ADAD2	adenosine deaminase domain containing 2	7.483	7.332	7.332	7.394	7.113
LINC01185	long intergenic non-protein coding RNA 1185	7.483	5.588	5.588	6.325	6.037
IGHV1-3	immunoglobulin heavy variable 1-3	7.483	7.152	7.152	7.055	7.547
NRBF2P2	nuclear receptor binding factor 2 pseudogene 2	7.483	7.054	7.054	6.476	7.416
TCP10/TCP10L2	t-complex 10	7.483	7.414	7.414	6.956	7.777
ABCA13	ATP binding cassette subfamily A member 13	7.410	7.827	7.827	7.316	7.928
XIAPP3	X-linked inhibitor of apoptosis pseudogene 3	7.410	6.573	6.573	3.292	6.835
TMEM26	transmembrane protein 26	7.332	6.709	6.709	6.158	6.491
BHLHE22	basic helix-loop-helix family member e22	7.332	7.702	7.702	6.736	7.928
ACAN	aggrecan	7.250	6.573	6.573	6.476	6.934
INSRR	insulin receptor related receptor	7.250	0	0	3.292	4.102
C6orf52	chromosome 6 open reading frame 52	7.163	4.871	4.871	0	4.659
SERPINA6	serpin family A member 6	7.163	6.709	6.709	6.476	7.416
HSPE1P4	heat shock protein family E (Hsp10) member 1 pseudogene 4	7.163	7.332	7.332	5.178	6.934
KRT8P31	keratin 8 pseudogene 31	7.163	5.846	5.846	6.476	6.355
MTND4P23	MT-ND4 pseudogene 23	7.071	3.381	3.381	5.968	5.63
TMEM26-AS1	TMEM26 antisense RNA 1	7.071	6.422	6.422	6.85	7.113
LINC01262	long intergenic non-protein coding RNA 1262	7.071	6.948	6.948	8.427	6.491
RPL23AP93	ribosomal protein L23a pseudogene 93	6.972	7.054	7.054	7.95	7.829
SERPINB7	serpin family B member 7	6.972	6.948	6.948	7.316	6.729
TEX41	testis expressed 41	6.972	6.422	6.422	6.956	7.416
C4orf45	chromosome 4 open reading frame 45	6.866	7.635	7.635	6.612	6.491
RSPO3	R-spondin 3	6.866	7.332	7.332	5.492	7.273
LINC00309	long intergenic non-protein coding RNA 309	6.866	6.833	6.833	7.148	7.608
HSD11B1	hydroxysteroid 11-beta dehydrogenase 1	6.866	4.310	4.310	4.217	6.355

CCDC116	coiled-coil domain containing 116	6.752	5.588	5.588	5.178	5.848
TMEM139	transmembrane protein 139	6.752	5.846	5.846	6.85	5.63
BARX1	BARX homeobox 1	6.752	0	0	5.178	4.102
SPATC1	spermatogenesis and centriole associated 1	6.752	7.766	7.766	6.85	6.934
LINC00589	long intergenic non-protein coding RNA 589	6.752	6.064	6.064	5.492	6.615
CKMT2	creatine kinase, mitochondrial 2	6.752	3.381	3.381	4.776	5.372

D.

Symbol	Entrez Gene Name	LPS	LEC10	LEC10+ LPS	LEC50	LEC50 + LPS
DMBX1	diencephalon/mesencephalon homeobox 1	-5.479	-5.417	-5.417	-2.2	-5.6
PGM5	phosphoglucomutase 5	-5.479	-5.417	-5.417	-5.492	-5.6
KANK4	KN motif and ankyrin repeat domains 4	-5.479	-1.107	-1.107	-2.2	-5.6
PCYT1B	phosphate cytidylyltransferase 1, choline, beta	-5.479	-5.417	-5.417	-1.275	-1.498
ANKRD33	ankyrin repeat domain 33	-5.479	-5.417	-5.417	-5.492	-2.416
FOXP1-AS1	FOXP1 antisense RNA 1	-5.479	0.171	0.171	-1.275	-0.541
ACY1	aminoacylase 1	-5.479	1.006	1.006	-2.2	-0.541
COLEC12	collectin subfamily member 12	-5.479	-5.417	-5.417	-2.2	-5.6
HHIP-AS1	HHIP antisense RNA 1	-5.479	-2.036	-2.036	-1.275	-0.941
FAM243A/FAM243B	family with sequence similarity 243 member A	-5.479	1.156	1.156	-0.716	-0.941
TRAV36DV7	T cell receptor alpha variable 36/delta variable 7	-5.479	1.156	1.156	0	0.755
RPS27AP12	ribosomal protein S27a pseudogene 12	-5.479	-2.036	-2.036	-1.275	-1.498
ZNRF2P1	zinc and ring finger 2 pseudogene 1	-5.479	1.416	1.416	-0.314	-5.6
RBP4	retinol binding protein 4	-5.479	0.838	0.838	0.666	-2.416
UTS2	urotensin 2	-5.479	-2.036	-2.036	-5.492	-1.498
PTTG4P	pituitary tumor-transforming 4 pseudogene	-5.479	-2.036	-2.036	0.257	-5.6
KAZN	kazrin, periplakin interacting protein	-5.479	-1.107	-1.107	-2.2	-0.941
DUTP1	deoxyuridine triphosphatase pseudogene 1	-5.479	-0.546	-0.546	-2.2	-2.416
TNFRSF17	TNF receptor superfamily member 17	-5.479	-0.546	-0.546	0.833	-2.416
TNNC2	troponin C2, fast skeletal type	-5.479	1.828	1.828	0.833	0.248
RPL13AP19	ribosomal protein L13a pseudogene 19	-5.479	-1.107	-1.107	-2.2	-5.6
BRCC3P1	BRCA1/BRCA2-containing complex subunit 3 pseudogene 1	-5.479	0.838	0.838	-1.275	-2.416
TRAJ14	T cell receptor alpha joining 14	-5.479	-2.036	-2.036	-0.716	-5.6
CYP7A1	cytochrome P450 family 7 subfamily A member 1	-5.479	-1.107	-1.107	-0.314	0.438
TEX21P	testis expressed 21, pseudogene	-5.479	-0.144	-0.144	-0.716	-0.941
TMC3-AS1	TMC3 antisense RNA 1	-5.479	-0.546	-0.546	-5.492	-5.6
TPI1P1	triosephosphate isomerase 1 pseudogene 1	-5.479	-5.417	-5.417	-0.314	0.03
LINC01629	long intergenic non-protein coding RNA 1629	-5.223	-5.16	-5.16	-1.018	-0.684
GDF6	growth differentiation factor 6	-5.223	-1.78	-1.78	-5.235	-2.159
ESYT3	extended synaptotagmin 3	-5.223	1.548	1.548	-1.018	-0.684
CA8	carbonic anhydrase 8	-5.223	-5.16	-5.16	-1.943	-2.159
SPATA3-AS1	SPATA3 antisense RNA 1 (head to head)	-5.223	-0.85	-0.85	-5.235	-1.241

AQP6	aquaporin 6	-5.223	-5.16	-5.16	-5.235	-2.159
CYP4F22	cytochrome P450 family 4 subfamily F member 22	-5.223	-5.16	-5.16	-5.235	-2.159
C1orf167	chromosome 1 open reading frame 167	-5.223	-5.16	-5.16	-5.235	-5.343
NT5C1B	5'-nucleotidase, cytosolic IB	-5.223	1.094	1.094	-0.46	-0.684
ELOVL3	ELOVL fatty acid elongase 3	-5.223	-0.85	-0.85	-1.018	-1.241
CAPN14	calpain 14	-5.223	-5.16	-5.16	-5.235	-0.684
DKFZp779M0652	uncharacterized DKFZp779M0652	-5.223	-1.78	-1.78	-0.058	-2.159
CD163L1	CD163 molecule like 1	-5.223	-0.85	-0.85	-1.018	-2.159
CCDC38	coiled-coil domain containing 38	-5.223	-1.78	-1.78	-5.235	-1.241
FHOD3	formin homology 2 domain containing 3	-5.223	-5.16	-5.16	-5.235	0.505
KLC3	kinesin light chain 3	-5.223	-5.16	-5.16	-5.235	-2.159
MFSD6L	major facilitator superfamily domain containing 6 like	-5.223	-0.85	-0.85	-1.943	-5.343
KIF4A	kinesin family member 4A	-5.223	-0.29	-0.29	-0.46	-0.684
UVRAG-DT	UVRAG divergent transcript	-5.223	-5.16	-5.16	-0.46	-5.343
MAGEB17	MAGE family member B17	-5.223	-5.16	-5.16	-5.235	-2.159
ACER1	alkaline ceramidase 1	-5.223	-1.78	-1.78	-1.018	0.03
LRFN2	leucine rich repeat and fibronectin type III domain containing 2	-5.223	-5.16	-5.16	-1.018	-0.284
LOC441204	uncharacterized LOC441204	-5.223	0.685	0.685	0.256	-0.684
OSBPL10-AS1	OSBPL10 antisense RNA 1	-5.223	-0.29	-0.29	0.514	0.695
TBX1	T-box 1	-5.223	-5.16	-5.16	-5.235	-2.159
STAB2	stabilin 2	-5.223	-5.16	-5.16	-5.235	-0.684
IQCJ-SCHIP1-AS1	IQCJ-SCHIP1 read through antisense RNA 1	-5.223	-5.16	-5.16	-5.235	0.03
CHRNA5	cholinergic receptor nicotinic alpha 5 subunit	-5.223	-0.29	-0.29	-1.018	-5.343
LINC00520	long intergenic non-protein coding RNA 520	-5.223	-0.85	-0.85	-1.943	0.03
DNMT3L	DNA methyltransferase 3 like	-5.223	-1.78	-1.78	-5.235	-5.343
PRRG2	proline rich and Gla domain 2	-5.223	-5.16	-5.16	-5.235	-0.284
NKX6-1	NK6 homeobox 1	-5.223	-5.16	-5.16	-0.46	-2.159
GRM4	glutamate metabotropic receptor 4	-5.223	-5.16	-5.16	-5.235	-2.159
CPB2	carboxypeptidase B2	-5.223	-1.78	-1.78	-0.058	-2.159
METTL24	methyltransferase like 24	-5.223	-1.78	-1.78	-5.235	-5.343
FERMT1	fermitin family member 1	-5.223	-5.16	-5.16	-5.235	-2.159
PARVA	parvin alpha	-5.223	-0.29	-0.29	-1.943	-0.284
RPL26P27	ribosomal protein L26 pseudogene 27	-5.223	-0.85	-0.85	-5.235	-2.159
TRAJ25	T cell receptor alpha joining 25 (non-functional)	-5.223	-5.16	-5.16	-1.018	-2.159
HRAT5	heart tissue-associated transcript 5	-5.223	-5.16	-5.16	-1.943	-1.241
HBQ1	hemoglobin subunit theta 1	-5.223	0.427	0.427	0.514	-1.241
NCR2	natural cytotoxicity triggering receptor 2	-5.223	-1.78	-1.78	1.24	0.695

*****These data represent the genes that are upregulated by LEC10 alone as compared to a vehicle control in PBMCs (incubated for 3h). RNAs were extracted and subjected to RNAseq analysis. This data has been obtained by using the DEG program. *****These data represent the genes that are upregulated by LEC10 alone as compared to a LPS control in PBMCs (incubated for 3h). RNA was extracted and subjected to RNAseq analysis. This data has been obtained by using the DEG program.

**These data represent the genes that are upregulated/downregulated by LPS, LEC10, LEC10 + LPS, LEC50 and LEC50 + LPS, as compared to vehicle controls in PBMCs (incubated for 3h). RNAs were extracted and subjected to RNAseq analysis. This data has been obtained by using the DEG program. The log2 ratios were imported into the Ingenuity Pathways Analysis.
